# A large and persistent outbreak of typhoid fever caused by consuming contaminated water and street-vended beverages: Kampala, Uganda, January – June 2015

**DOI:** 10.1186/s12889-016-4002-0

**Published:** 2017-01-05

**Authors:** Steven Ndugwa Kabwama, Lilian Bulage, Fred Nsubuga, Gerald Pande, David Were Oguttu, Richardson Mafigiri, Christine Kihembo, Benon Kwesiga, Ben Masiira, Allen Eva Okullo, Henry Kajumbula, Joseph Matovu, Issa Makumbi, Milton Wetaka, Sam Kasozi, Simon Kyazze, Melissa Dahlke, Peter Hughes, Juliet Nsimire Sendagala, Monica Musenero, Immaculate Nabukenya, Vincent R. Hill, Eric Mintz, Janell Routh, Gerardo Gómez, Amelia Bicknese, Bao-Ping Zhu

**Affiliations:** 1 Uganda Public Health Fellowship Program, Field Epidemiology Track, Ministry of Health, Kampala, Uganda; 2 Makerere University College of Health Science Microbiology Laboratory, Kampala, Uganda; 3 Makerere University School of Public Health, Kampala, Uganda; 4 Public Health Emergency Operations Center, Ministry of Health, Kampala, Uganda; 5 Medical Research Council, Kampala, Uganda; 6 Epidemiology and Surveillance Division, Ministry of Health, Kampala, Uganda; 7 US Centers for Disease Control and Prevention, Atlanta, Georgia USA; 8 US Centers for Disease Control and Prevention, Kampala, Uganda

**Keywords:** Typhoid fever, Outbreak, Case-control, Uganda

## Abstract

**Background:**

On 6 February 2015, Kampala city authorities alerted the Ugandan Ministry of Health of a “strange disease” that killed one person and sickened dozens. We conducted an epidemiologic investigation to identify the nature of the disease, mode of transmission, and risk factors to inform timely and effective control measures.

**Methods:**

We defined a suspected case as onset of fever (≥37.5 °C) for more than 3 days with abdominal pain, headache, negative malaria test or failed anti-malaria treatment, and at least 2 of the following: diarrhea, nausea or vomiting, constipation, fatigue. A probable case was defined as a suspected case with a positive TUBEX® TF test. A confirmed case had blood culture yielding *Salmonella* Typhi. We conducted a case-control study to compare exposures of 33 suspected case-patients and 78 controls, and tested water and juice samples.

**Results:**

From 17 February–12 June, we identified 10,230 suspected, 1038 probable, and 51 confirmed cases. Approximately 22.58% (7/31) of case-patients and 2.56% (2/78) of controls drank water sold in small plastic bags (OR_M-H_ = 8.90; 95%CI = 1.60–49.00); 54.54% (18/33) of case-patients and 19.23% (15/78) of controls consumed locally-made drinks (OR_M-H_ = 4.60; 95%CI: 1.90–11.00). All isolates were susceptible to ciprofloxacin and ceftriaxone. Water and juice samples exhibited evidence of fecal contamination.

**Conclusion:**

Contaminated water and street-vended beverages were likely vehicles of this outbreak. At our recommendation authorities closed unsafe water sources and supplied safe water to affected areas.

**Electronic supplementary material:**

The online version of this article (doi:10.1186/s12889-016-4002-0) contains supplementary material, which is available to authorized users.

## Background

Typhoid fever is a systemic disease caused by *Salmonella enterica* serovar Typhi, a Gram-negative bacterium. Humans are the only host, and transmission most commonly occurs through ingestion of water or food contaminated by feces from an acutely ill or convalescent patient or an asymptomatic carrier. The incubation period is usually 1 to 2 weeks but can range from 3 to 60 days [1]. The illness presents with sustained fever and a constellation of other symptoms including dry cough, fatigue, abdominal pain, diarrhea, and constipation [2]. Case fatality ratios range between 10 and 30% if untreated, but fall to 1–4% with appropriate and timely antimicrobial treatment [3]. The gold standard laboratory diagnosis of typhoid fever requires isolation of *S.*. Typhi from blood, stool, bone marrow, or other tissue or bodily fluid by bacterial culture [2]. Other tests with moderate sensitivity and specificity include the Widal test and TUBEX® TF test which involve detection of antibodies against *S.* Typhi antigens [2]. Typhoid fever is preventable through public health interventions such as provision of safe water, ensuring proper sanitation and waste disposal systems, and excluding disease carriers from handling food [4].

Typhoid fever is a major cause of mortality and morbidity worldwide. In endemic areas, the disease is most commonly found in children 5–19 years of age. International visitors from non-endemic areas are also at risk if unvaccinated [1]. The global burden of the disease in low- and middle-income countries in 2010 was estimated to be 11.9 million cases, including 129,000 fatalities, after adjusting for water-related risk factors [5]. In Uganda, an outbreak of typhoid fever in Kasese District sickened 8092 persons from 27 December 2007 to 30 July 2009, resulting in at least 249 intestinal perforations and 47 deaths [6]. In 2011, numerous typhoid cases were again reported in Kasese and neighboring Bundibugyo District with many more intestinal perforations and emergence of multidrug resistant strains [7].

On 6 February, 2015, the Ugandan Ministry of Health (MoH) received a report from the Kampala Capital City Authority that a 42-year-old man had died a day earlier of a “strange illness.” The patient was admitted to the hospital on 2 February 2015 with symptoms of abdominal pain, high fever, and severe jaundice. Initial testing involved use of the Widal test which was positive. Approximately 30 other people who worked in the same area as the deceased reportedly had similar symptoms. We conducted an epidemiologic investigation to identify the nature of the disease, mode of transmission, and risk factors to inform timely and effective control measures.

## Methods

### Study sites

The outbreak occurred in Kampala (estimated population: 1.4 million), the capital of Uganda [8]. Kampala has five divisions: Kampala Central, Kawempe, Makindye, Rubaga, and Nakawa. We focused our epidemiologic investigation on two markets and a commuter taxi park in Kampala Central Division where the initial cases were concentrated.

### Surveillance

To characterize and control the epidemic, MoH conducted surveillance at six treatment centers established in affected areas of the city to provide diagnostic testing and typhoid fever treatment free of charge. These treatment centers were existing health centers in which routine disease surveillance and treatment activities are conducted. Through the media, local leaders encouraged the people with symptoms of typhoid fever to seek medical care at these treatment centers.

We defined a suspected case as onset of fever (≥37.5 °C) for ≥3 days from 1 January 2015 onwards, with headache, abdominal pain, a negative test for malaria or failure to respond to anti-malaria treatment, and ≥2 of the following symptoms: diarrhea, nausea or vomiting, constipation, or fatigue. A probable case was a suspected case whose serum sample yielded a positive TUBEX® TF test [9]. Blood samples were collected from the first 5 suspected cases every day from each treatment center and referred to the microbiology laboratory at the Medical Research Council for blood culture. A confirmed case was a suspected case whose blood culture yielded *S*. Typhi.

### Case-control study

We conducted open-ended hypothesis-generating interviews of case-patients found in the areas where the initial cases were identified, focusing on their usual sources of water and food. To test the hypotheses generated from the interviews, we conducted a case-control study from 10 to 20 February 2015. To rapidly identify the mode of transmission so as to inform prompt prevention and control measures, we used the initial 33 suspected case-patients identified in the earliest-affected communities for the case-control study. The earliest cases were persons working in two markets or in the central terminal station for Kampala’s shared taxis, all of which were located in central Kampala. Therefore we recruited both the cases and the controls from those places. The markets are open spaces where people set up their stalls to sell assorted merchandise, whereas the central terminal station for the shared taxis is an area where the shared taxis (mini-vans) pick up and drop off passengers. In the markets, after identifying and interviewing a case, the interviewer then walked around the stall to identify several persons of the same gender and similar age as the case from the surrounding stalls who never had a febrile disease since January 1, 2015, and recruited those persons as controls. Similarly, in the central terminal station for the shared taxis, after identifying and interviewing a case who was working inside a shared taxi (e.g., a driver or conductor), the interviewer then walked around the shared taxi to recruit asymptomatic workers of the same gender and similar age from the surrounding shared taxis as controls. The interviewers used a structured questionnaire to collect information on the usual water and food exposures from the case- and control-persons. A link to the questionnaire that was used has been provided in the Additional file 1 of the manuscript.

### Clinical laboratory investigation

The TUBEX® TF test was performed at the treatment centers by trained clinical and laboratory staff as per the manufacturer’s instructions. Blood culture was performed on the first five patients presenting each day at the 6 treatment centers. From each adult patient, 5–10 mL of blood was collected and inoculated in BD Bactec™ Aerobic/F blood culture bottles and incubated in a BD Bactec 9000 series™. Presumptive positive bottles, as signaled by the system, were subcultured on MacConkey, chocolate, and blood agar plates and incubated aerobically at 37 °C for 24 h. A Gram stain was also performed. Negative vials were incubated for up to 7 days and if the system still indicated negative, a Gram stain was performed and a final subculture was done before reporting the specimen as negative. Oxidase-negative, lactose non-fermenting colonies, were screened using API 10S at the start of the outbreak. Later an abbreviated panel of biochemical tests [10] was used. Isolates biochemically typical of *S.* Typhi were serotyped using slide agglutination with *S.* polyvalent O, *S.* polyvalent H, *S.* O factor 9 (group D), *S.* H factor d and *S.* Vi antisera.

A set of 30 *S*. Typhi isolates were sent to the U.S. Centers for Disease Control and Prevention (CDC) for confirmation and antimicrobial susceptibility testing (AST). The National Antimicrobial Resistance Monitoring System at CDC performed AST on 17 isolates by broth microdilution to determine minimum inhibitory concentrations for 14 antimicrobial agents: amoxicillin/clavulanic acid, ampicillin, azithromycin, ceftiofur, ceftriaxone, cefoxitin, chloramphenicol, ciprofloxacin, gentamicin, nalidixic acid, streptomycin, sulfisoxazole, tetracycline, and trimethoprim/sulfamethoxazole. Results were interpreted using Clinical and Laboratory Standards Institute standards [11] when available.

During the case-control study, we collected 5–10 mL of blood from each of 20 suspected case-patients, placed the samples into commercial BD Bactec™ Aerobic/F media, and transported them to the clinical laboratory at the Makerere College of Health Sciences Department of Medical Microbiology for incubation in the Bactec 9120™ blood culture system. Subcultures onto MacConkey and blood agar were done following instrument signals of growth or at the end of 7 days of incubation. Colonies were identified as *S.* Typhi based on biochemical characteristics including motility, hydrogen sulfide production, fermentation of sugars, urease production, and serological typing characteristics with various specific antisera.

### Environmental laboratory investigation

From 2 to 8 April, juice samples were collected from the Nakasero, Owino, and Shauriyako markets, and 100 mL water samples were collected from unprotected water sources such as unprotected springs (i.e., underground water sources that do not have barriers protecting them from contamination and run-off) and commercial vendors in Kampala Central Division. We chose these water collection sites because we observed people in the outbreak-affected areas collecting water from these sites. The juice samples were tested because case-persons said they usually consumed these drinks. We collected nine juice samples, including 3 “bushera” (millet and yeast), 2 “munanansi” (pineapple juice with tea leaves), 3 “butunda” (passion fruit), and 1 “bongo” (unpasteurized yogurt drink). We also collected 13 water samples, including 3 “kaveera” (water packaged and sold in a small plastic bag), one unlabeled bottle of water from a street vendor, water from three storage tanks, and water from five unprotected springs. Juice and water samples were tested using a modified version of the United States Environmental Protection Agency’s Standard Analytical Protocol for *S.* Typhi in Drinking Water [12]. Briefly, 125 mL of specimen was pre-enriched in 125 mL of double strength buffered peptone water at 37 °C, followed by parallel enrichment in Selenite Cysteine broth at 37 °C and RV broth at 42 °C. Cultures from Selenite Cysteine broth were plated onto MacConkey and XLD agars; cultures from RV broth were plated onto XLD agar. All plates were incubated at 37 °C. Plates were inspected at 24 and 48 h for colony morphology consistent with *Enterobacteriacea.* Colonies morphologically consistent with *S.* spp. (i.e. lactose negative) were subjected to biochemical testing. Suspect isolates were sent to CDC-Atlanta for biochemical confirmation. For confirmation, suspect cultures were streaked onto Hektoen enteric agar and suspect colonies were subjected to an abbreviated panel of tests, for phenotypic identification of *Salmonella* or *Shigella* spp. and biochemical differentiation of *S.* serovars Typhi and Paratyphi A from other *Salmonella* serovars [10].

### Statistical analysis

Using surveillance data, the attack rates by sex, division, and sex were calculated using population data from the national census [8] and data provided by the Uganda Bureau of Statistics [13]. Using the StatCalc in Epi Info 7, considering a power of 80%, two sided confidence level of 95%, a case-control ratio of 1:2 with 30% of cases exposed and 10% of controls exposed, we would require about 39 cases and 77 controls. To measure the associations between exposure variables and illness status, we used the Mantel-Haenszel method to estimate odds ratios (OR) and their confidence intervals, accounting for frequency-matching of cases and controls. We calculated the proportion of cases and controls who drank 1, 2, and 3–4 types of locally made drinks, and used the Chi-square test for linear trend to assess the relationship between the number of types of drinks consumed and odds of illness [14].

## Results

### Surveillance

**Fig. 1:**
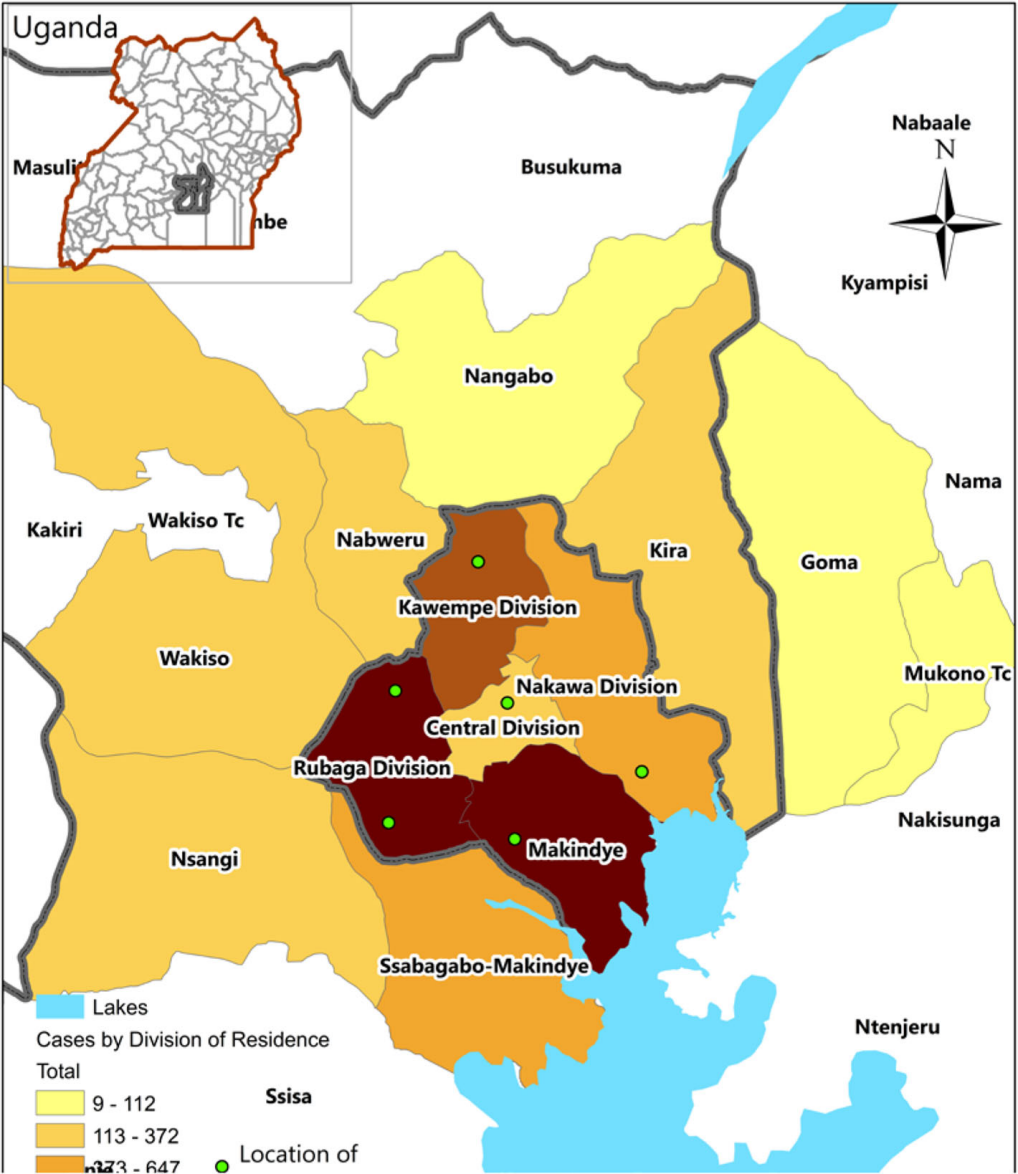
An intensity map showing treatment centers (*green dots*) and geographical distribution of suspected case-patients by place of residence during an outbreak of typhoid fever: Kampala, Uganda, 17^th^ February 2015 – 14^th^ April 2015 (*n* = 9325)

From 17 February to 12 June 2015, we identified 10,230 suspected cases from the six treatment centers established by MoH. Cases were distributed widely throughout Kampala and neighboring areas (Fig. 1).

**Fig. 2:**
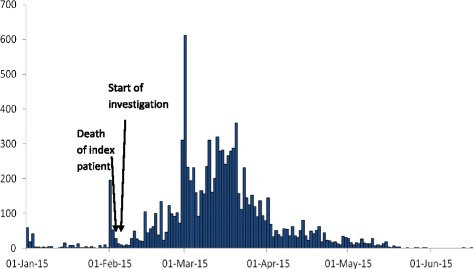
Distribution of suspected cases of typhoid fever by date of symptom onset during an outbreak of typhoid fever: Kampala, Uganda, 1 January 2015-12 June 2015 (*n* = 9515)*

The epidemic curve of suspected cases suggests that the outbreak started at the beginning of February or perhaps earlier. By the time the outbreak was recognized on 6 February, hundreds of cases had already occurred (Fig. 2). Cases were reported in all five divisions of Kampala: Makindye (32%, 3234), Rubaga (28%, 2828), Kawempe (11%, 1144), Nakawa (6.4%, 656) and Central (4.2%, 428); for 19% (1940) of cases, either no division of residence was identified, or resided outside of Kampala. The attack rate during the outbreak period was highest in Makindye (10/1,000), Rubaga (8.7/1000), and Central (6.5/1000) Divisions. Males had a higher attack rate than females. The attack rate among people in the 15–59 year age group (12/1000) was 6 times higher than among younger (2.0/1000) or older (2.0/1000) persons (Table 1).

**Table 1:** Attack rate^a^ (per 1000 residents) by sex, age group, and Division of residence during an outbreak of typhoid fever: Kampala, Uganda, 17 February 2015–12 June 2015

Characteristic	Attack rate (/1000 residents)
Sex	
Male	11.00
Female	8.40
Total	9.50
Age group (years)	
<15	2.00
15–59	12.00
≥60	2.00
Division of residence^a^	
Central	6.50
Kawempe	3.80
Makindye	10.00
Rubaga	8.70
Nakawa	2.30

^a^ Based on suspected cases

### Case-control study

In our hypothesis-generating interviews of patients from the area where the outbreak was first identified, consumption of drinks made with water extracted from unprotected sources and packed in unhygienic conditions was often reported.

Of the 33 case-patients we enrolled in the case-control study, 60% were men; the majority of the case-patients (85%) were in the 20–39 year and 9.1% in older age groups. In addition to fever, commonly reported symptoms included abdominal pain (72.72%) and headache (69.69%) (Table 2). We found that 22.58% (7/31) of case-patients compared with 2.56% (2/78) of controls usually drank locally packaged water in small plastic bags called *“*kaveera water*”* (OR_M-H_ = 8.90; 95% CI = 1.60–49.00); 55% (18/33) of case-patients compared with 19.23% (15/78) of controls drank locally-made passion fruit juice called *“*butunda*”* (OR_M-H_ = 4.60; 95% CI: 1.90–11.00); 31.25% (10/32) of case-patients compared with 16.67% (13/78) of controls usually drank locally-packed pineapple juice called *“*munanansi*”* (OR_M-H_ = 2.00; 95% CI = 0.74–5.20); and 15.63% (5/32) of case-patients compared with 8.97% (7/78) of controls usually drank cold millet porridge called *“*bushera*”* (OR_M-H_ = 2.80; 95% CI = 0.76–10.00). Workplace as a source of breakfast (OR_M-H_ = 0.25; 95% CI = 0.07–0.93), and workplace as a source of lunch (OR_M-H_ = 0.35; 95% CI = 0.11–1.10) were not significant risk factors for illness.

**Table 2:** Reported symptoms among case-patients in a case-control study during an outbreak of typhoid fever: Kampala, Uganda, 10 February 2015-20 February 2015 (*n* = 33)

Symptom	Frequency	Percent
Fever ≥3 days^a^	33	100
Abdominal Pain	24	72.72
Headache	23	69.69
Diarrhea	10	30.30
Nausea or Vomiting	10	30.30
Jaundice	7	21.21
Constipation	3	9.09

^a^ Since 1 January, 2015

When we compared the proportions of case-patients and controls who drank 0, 1, 2, or 3–4 types of locally-made drinks, we found that case-patients were more likely to drink multiple types of locally-made drinks than controls (Chi-square for linear trend = 14.65, *p* < 0.001) (Table 3).

**Table 3:** Dose-response relationship comparing those who consumed 1, 2 and 3–4 implicated drinks to those who did not consume any of the drinks during an outbreak of typhoid fever: Kampala, Uganda, February 2015

Drinks	%Cases (*n* = 33)	%Controls (*n* = 78)	OR
0	36	64	1.0 (Ref)
1	27	26	1.90 (0.68–5.10)
2	15	9.0	3.00 (0.80–11.00)
3–4	21	1.0	29.00 (3.20–260.00)

### Laboratory investigation

Of 10,230 suspected cases, 3464 (10%) underwent TUBEX® TF testing. Of those, 1038 were positive, representing a positivity rate of 29%. Blood samples from a total of 364 patients (including 20 of 33 case-control study patients) were tested by blood culture and 56 (15%) (including 5 of the 20 case-control study patients tested) yielded *S. enterica* ser. Typhi.

Subsequently, 30 of the 56 *S*. Typhi isolates from blood cultures were confirmed at the U.S. CDC as *S*. Typhi. CDC determined the minimum inhibitory concentrations for 17 of these isolates, 5 of which were resistant to ampicillin, chloramphenicol, streptomycin, sulfisoxazole, nalidixic acid, trimethoprim/sulfamethoxazole and had intermediate interpretation to ciprofloxacin. The remaining 12 were resistant to nalidixic acid and had intermediate interpretation to ciprofloxacin.

### Environmental investigation

One of 3 “kaveera water” samples and the unlabeled bottle of water sold by street vendors contained lactose fermenting bacteria, which are commonly *Enterobacteriaceae* and associated with fecal contamination. The 2 other “kaveera water” samples contained non-lactose fermenting bacteria, also consistent with fecal contamination, and one isolate was further identified as non-typhoidal *Salmonella*. The 5 water samples from unprotected springs showed evidence of robust contamination with lactose fermenting bacteria. Lactose non-fermenting colonies from 2 spring water samples were identified as non-typhoidal *Salmonella* spp. Lactose fermenting bacteria were also detected in 2 of 3 “bushera” samples, 1 of 2 “munanasi” samples, all 3 “munanansi” juice samples, and the “bongo” sample. Additionally, non-typhoidal *Salmonella* was cultured from 1 “bushera”, 1 “munanansi”, and 1 passion fruit juice sample.

## Discussion

Our investigation revealed a prolonged and widespread outbreak of typhoid fever that affected thousands of people in all five divisions of Kampala City over several months. Contaminated water from unprotected sources and drinks made with it were the likely vehicles of infection early in the outbreak. Juice and water samples obtained from street vendors and water samples collected from unprotected spring water sources showed evidence of fecal contamination. Although *S*. Typhi was not recovered from environmental testing, non-Typhi *Salmonella* were isolated from five street-vended beverage samples from the implicated markets.

All 17 isolates of *S*. Typhi from blood tested at CDC were resistant to nalidixic acid and had intermediate interpretation to ciprofloxacin. It is possible that persons affected by the antibiotic-resistant strains during this outbreak had experienced complications of typhoid fever considering that antibiotic-resistant strains of *S.* Typhi are associated with more severe form of the illness, complications and death [15].

This outbreak may have started in January 2015 or even earlier; however, it was not recognized until early February because routine clinical and laboratory surveillance systems for typhoid fever were not in place before the investigation. The widespread nature of the outbreak is compatible with a waterborne source. The sudden increase in cases after the start of the investigation was likely due to active community outreach and education about the symptoms of typhoid fever and the availability of prompt, free diagnostic testing and treatment through newly established treatment centers. The gradual decline in cases from mid-March onwards was likely the result of patient treatment and public health interventions including provision of free water chlorination products, sensitization of residents on water treatment, and the establishment of free alternative safe water sources in the most affected communities. Based on the evidence we presented, the Kampala Capital City Authority sealed off all underground water sources and worked with the National Water and Sewerage Corporation to ensure the provision of accessible alternative sources of water to the affected communities.

In Uganda, as in many low and middle income countries, definitive diagnostic tests for typhoid fever such as blood culture are usually unavailable, unaffordable, or inconsistently applied [16]. Instead, typhoid fever diagnosis and surveillance often rely on clinical judgment or on the Widal test, which has poor sensitivity and specificity [17]. Moreover, physicians often give presumptive antibiotic and/or antimalarial treatment for febrile illnesses [18, 19] without attempting to determine the etiology. Previous studies have indicated that a significant proportion of febrile illness in Uganda is caused by bacteremia, including invasive non-Typhi salmonellosis and typhoid fever [20]. A more robust approach in these settings could entail periodically identifying persons with febrile illness in the communities and taking blood culture for confirmation [21]. The blood samples could be collected and sent using a specialized transport network to regional laboratory centers around the country where confirmative tests can be performed. This system has been successfully used to improve diagnostic services in early infant HIV/AIDS diagnosis [22]. Sentinel surveillance for febrile illnesses based on blood cultures would accelerate the early identification of outbreaks and implementation of control measures.

Waterborne typhoid and paratyphoid fever affect an estimated 27 million people worldwide each year [3]. In developing countries, where safe water and sanitation systems have not been well-established, large-scale typhoid and paratyphoid outbreaks sometimes occur [7, 23–26]. During a previous typhoid outbreak in Kasese and Bundibugyo districts, Uganda, in 2009–2011, which affected 8092 persons, the vehicle of transmission was also found to be unclean water [7]. The current outbreak was likely caused by consuming contaminated water from unprotected ground water sources. Kampala city has more than 200 unprotected ground water sources, most of which serve as unprotected sources of water for economically disadvantaged people in the city such as those in our investigation [27]. Unsafe disposal of excreta and solid waste are significant factors that contribute to contamination of ground water in Kampala [28]. This outbreak investigation highlights the importance of ensuring access to affordable, safe, treated drinking water and improved sanitation and waste management systems for resource-constrained urban populations.

Risk factors for typhoid transmission were not assessed later during this outbreak, when foodborne transmission might have become more common. Recurrent contamination of unprotected water sources with *S*. Typhi likely continued to sustain the outbreak propagation over the course of several months.

According to the Uganda Demographic Health Survey 2011 [29], almost 30% of people living in urban areas and more than 60% of those living in rural areas do not treat their water before drinking it. Barriers to safer drinking water include the cost associated with establishing a piped treated water system or purchasing water treatment products for household use and the false perception that naturally occurring water sources could be safe [30].

In the aftermath of outbreaks like this one, public health authorities face 3 possible options: The first option is to do nothing but respond to outbreaks as they occur. Governments in resource-constrained settings often choose this option, leaving the population vulnerable to outbreaks of waterborne diseases including cholera, hepatitis A and E, cryptosporidiosis, shigellosis, and many others in addition to typhoid and paratyphoid fever. The second option is mass vaccination against typhoid fever. A cost-effectiveness evaluation of a hypothetical typhoid vaccination campaign was carried out after the multi-year outbreak of typhoid fever in Kasese District, Uganda, and it was estimated to be highly cost-effective [31]. However, vaccination against the many different pathogens that cause waterborne diseases is not possible because vaccines are not yet available for many of them (e.g. cryptosporidiosis, shigellosis, paratyphoid fever, etc.). In addition, typhoid fever vaccines have been shown to have varied levels of effectiveness (from 50 to 95%) and to last for varied lengths of time (from 3 to 10 years) [15].

The third and final option is to improve the water and sanitation systems. Improvement of sanitation, hygiene and clean water supply around the world could avert ≥90% of diarrheal disease episodes annually [32]. In North America and Europe, typhoid fever caused large-scale outbreaks from the late 19^th^ through the early 20^th^ century [33, 34]. After improvement of municipal water and sanitation systems in the early 20^th^ century, waterborne outbreaks of communicable diseases including typhoid fever drastically decreased [35–37]. Improved sanitation measures such as having a basic pit latrine or a toilet connected to a septic tank curtail the direct contact between human waste and water or the environment. Yet in 2012, only 33% of the urban population in Uganda had access to adequate sanitation, an increase of only 1% since 1990, and 2% still practiced open defecation [38]. Although improving water and sanitation systems requires a substantial investment by the government, ultimately it is highly cost-effective in the reduction of many waterborne diseases [39].

### Strengths and limitations

A major limitation of our investigation was that, due to inadequate laboratory capacity to confirm a large number of cases early in the outbreak, and the need to rapidly identify the mode of transmission to inform effective interventions, we included non-laboratory confirmed cases in our case-control study. While a clinical case definition for typhoid fever cases can lead to misclassification, and is not recommended during non-outbreak situations, during an outbreak such a case definition will often perform well, as measured by good positive and negative predictive values [40]. Also, the information on usual sources of water and food was based on self-reports, which could represent a source ofinformation bias. Another limitation is that data on mortality or on complications such as intestinal perforations were not collected. With over 10,000 cases it is likely that there were intestinal perforations and deaths but no surveillance for those outcomes was done. A study is currently being conducted to assess these severe impacts of this outbreak. In addition, only a few water and juice samples were tested, which could explain why *S.* Typhi was not isolated during the environmental investigation. Also, water and juice samples were tested using the reagents and procedures that were available in the laboratory for testing clinical specimens for Salmonella, and not more conventional methods for evaluating the potential presence of fecal contamination in these types of samples.

## Conclusion

In conclusion, this investigation revealed a large outbreak of typhoid fever that affected thousands of people in Kampala, Uganda, which appeared to have been caused by consuming contaminated water and local drinks made from it. To prevent future waterborne outbreaks, we recommended that the Kampala Capital City Authority, the MoH, the National Water and Sewerage Corporation, and partners invest in improving access to potable water, and safe sanitation and hygiene facilities [41].

## Additional file

Additional file 1: The questionnaire that was used in the collection of information from cases and controls is available as a supplementary file called Typhoid questionnaire. It provides details on which questions were asked about food and water exposures. (DOC 87 kb)

## Abbreviations

CDC: United States Centers for Disease Control and Prevention
MoH: Ministry of Health
WHO: World Health Organization
