# Correction to: A large and persistent outbreak of typhoid fever caused by consuming contaminated water and street-vended beverages: Kampala, Uganda, January – June 2015

**DOI:** 10.1186/s12889-017-4801-y

**Published:** 2017-10-18

**Authors:** Steven Ndugwa Kabwama, Lilian Bulage, Fred Nsubuga, Gerald Pande, David Were Oguttu, Richardson Mafigiri, Christine Kihembo, Benon Kwesiga, Ben Masiira, Allen Eva Okullo, Henry Kajumbula, Joseph KB Matovu, Issa Makumbi, Milton Wetaka, Sam Kasozi, Simon Kyazze, Melissa Dahlke, Peter Hughes, Juliet Nsimire Sendagala, Monica Musenero, Immaculate Nabukenya, Vincent R. Hill, Eric Mintz, Janell Routh, Gerardo Gómez, Amelia Bicknese, Bao-Ping Zhu

**Affiliations:** 1 Uganda Public Health Fellowship Program, Field Epidemiology Track, Ministry of Health, Kampala, Uganda; 2 Makerere University College of Health Science Microbiology Laboratory, Kampala, Uganda; 3 Makerere University School of Public Health, Kampala, Uganda; 4 Public Health Emergency Operations Center, Ministry of Health, Kampala, Uganda; 5 Medical Research Council, Kampala, Uganda; 6 Epidemiology and Surveillance Division, Ministry of Health, Kampala, Uganda; 7 US Centers for Disease Control and Prevention, Atlanta, GA USA; 8 US Centers for Disease Control and Prevention, Kampala, Uganda

After publication of the article [1], it has been brought to our attention that the author Joseph Matovu is missing his initials from his full professional title ‘Joseph KB Matovu’.
