# Supplementary material for: A large and persistent outbreak of typhoid fever caused by consuming contaminated water and street-vended beverages: Kampala, Uganda, January – June 2015
Source: BMC Public Health. 2017 Jan 5;17:23. doi: 10.1186/s12889-016-4002-0 (PMC5216563; doi:10.1186/s12889-016-4002-0)
Supplement: Additional file 1: — The questionnaire that was used in the collection of information from cases and controls is available as a supplementary file called Typhoid questionnaire. It provides details on which questions were asked about food and water exposures. (DOC 87 kb) [file 12889_2016_4002_MOESM1_ESM.doc]

Appendix 1

***KCCA /MakSPH/MoH***

**QUESTIONNAIRE FOR INVESTIGATION OF TYPHOID FEVER OUTBREAK IN KAMPALA CENTRAL BUSINESS DISTRICT**

On Thursday 5^th^ February 2015, the medical department Kampala City Council Authority (KCCA) received information from your Local Council Authorities that an unknown illness had occurred around your area and it had affected several people.

The KCCA team immediately contacted the ministry of health which has formed a team to investigate the cause of the problem in this area. You have been identified as one of the people affected by this disease and I would like to ask you a few questions about how the illness has affected you. This information is very important and shall be used to get a solution to this problem. I want to assure you that the information you provide shall be kept confidential.

The purpose of this study is to establish the most likely source of the outbreak in order to implement control measures.

**DEMOGRAPHIC DATA**

ID NO

|  | SOURCE OF DATA:  Record review  Primary |
| --- | --- |

NAME: ……………………………………………………………………………………………………………………………………….

TEL. CONTACT …………………………………………………………………………………………………………………………..

| AGE | GENDER:  M  F |
| --- | --- |

PLACE OF RESIDENCE ………………………………………………………………………………………………………………..

PLACE OF WORK ……………………………………………………………………………………………………………………….

WORKING IN CENTRAL DIVISION: Yes No

OCCUPATION ……………………………………………………………………………………………………………………………

**CLINICAL DATA**

*Please tick* ***Yes*** *or* ***No*** *to any of the following questions:*

1 (a) have you had fever since 01/Jan/2015?

Yes

No

(b) If **Yes**, indicate date of onset (DD/MM/YY):

|  |  |  |  |  |  |
| --- | --- | --- | --- | --- | --- |

How many days did you have the fever?

|  |  |  | days |
| --- | --- | --- | --- |

2) Have you also had the following complaints?

| **Symptom** | **Yes** | **No** |
| --- | --- | --- |
| Headache |  |  |
| Abdominal pain |  |  |
| Diarrhea |  |  |
| constipation |  |  |
| Nausea/vomiting |  |  |
| Altered mental state |  |  |
| Yellow eyes |  |  |
| Other complaints (specify) |  | |

N

Don’t Know

Y

4) During the current illness, have you used any antimalarials?

Y

N

Don’t Know

Have you used anti biotics?

Any Other Drugs?.......................................................................................................................................

**Food and drinks intake at the workplace:**

1. While at work, where do you usually get your drinking water from?
   - Nakasero Market
   - Disableds Market
   - New Park
   - Super market
   - Shops
   - Home
   - Other…………………………………………………………………………………………………………………..
2. While at work, what type of water do you usually drink?
   - Bottled (sealed)
     1. Which brand and quantity? ..................................................
     2. How much does this water cost? ..........................................
     3. Approximately how much of this water do you drink in a day?
   - Bottled (not sealed)
     1. How much does this water cost? ..................................................
     2. Approximately how much of this water do you drink in a day?

..................................................

- - Water packed in kaveera – polyethene.
    1. How much does this water cost? ..................................................
    2. Approximately how many buveera of water do you drink in a day?

..................................................

- Not Packed **AND** Not bottled

1. Besides water, which other drinks do you take while at work?
   - Cold Bushera
   - Juice
     1. Pineapple (munanansi)
     2. Passion fruit (butunda)

Any other drinks (Specify) ……………………………………………………..

1. Where do you get these drinks from? ………………………………………………..
2. What type of fruits do you eat while at work?
   - Packed fruits
   - Unpacked (specify) ……………………………………………………….
   - None
3. What foods do you usually eat while at work?
   - Breakfast………………………………………………………………………………………………………..
     1. Where do you usually get the food from? ……………………………………………….
   - Lunch ……………………………………………………………………………………………………………..
     1. Where do you usually get the food from? ...................................................
   - Other……………………………………………………………………………………………………………….
     1. Where do you usually get the food from? ……………………………………………….

Any other comments

…………………………………………………………………………………………………………………………………………………………

**Thank you**

Appendix 2:

# KCCA/MUSPH CASE INVESTIGATION LABORATORY REQUEST FORM

Date…………………………………………………………………………………………………………………………………………………………

Name ……………………………………………………………….sex…………….age……………………………ID……………………………..

Name of Reference Laboratory………………………………………………………………………………………………………………..

Sample 1:…………………………………………………..Test requested………………………………………………………………………

………………………………………………………………………………………………………………………………………………………..………..

Sample 2: ………………………………………………………Test requested……………………………………………….…………………

......................................................................................................................................................................

**Clinical notes:**

**Laboratory report:**
